# Building international infection prevention and control capacity during COVID-19: Case studies from Ethiopia and Indonesia

**DOI:** 10.7189/jogh.13.03020

**Published:** 2023-05-12

**Authors:** Hailey Bednar, Ramot Adeboyejo, Turquoise Sidibe, Rachel Powell

**Affiliations:** CDC Foundation, Atlanta, Georgia, USA

Investing in preparedness is essential to healthcare resiliency, yet the COVID-19 pandemic exposed the inefficiencies in many healthcare systems and processes. Defined as the ability to resist, tolerate, recover from, prepare for or adapt to an adverse event that causes harm, destruction or loss, healthcare resiliency reflects the ways in which healthcare can face unexpected overloading without degradation of healthcare delivery [1].

Health system resiliency has been previously defined as “the capacity of health actors, institutions and populations to prepare for and effectively respond to crises; maintain core functions when a crisis hits, and; be informed by lessons learnt during the crisis and reorganize if conditions require it” [2,3]. To provide equitable access to care and promote healthy societies, healthcare systems must work alongside communities to develop resilience according to their needs, emphasising approaches that engage relevant local, regional, and national health authorities, and committing to clear coordination beyond responding to infectious disease outbreaks [4].

Infection prevention and control (IPC) is defined as a “practical, evidence-based approach which prevents patients and health workers from being harmed by avoidable infection and as a result of antimicrobial resistance” [5]. A scoping review of recurring themes and capacities showed that resilient health systems have strong IPC measures, including staff training, standardized protocols and dedicated treatment units [6].

While poor IPC is linked to infectious disease transmission among patients and healthcare workers, a lack of national IPC strategies or dedicated leaders at facility or national levels is associated with inconsistent messaging and coordination, leading to poor response coordination and the spread of misinformation in communities [7]. Investing in and maintaining IPC helps strengthen interactions between infectious disease-specific programmes and patient access to care [8], consequently strengthening healthcare systems.

The following case studies discuss how two international grantee programmes supported by the National Foundation for the Centers for Disease Control and Prevention (CDC Foundation) used designated flexible funding to improve and strengthen the grantee countries’ IPC systems.

## Case study: IPC assessment and strengthening in Ethiopia

In March 2020, Ethiopia reported its first COVID-19 case [9], responding by introducing a series of multisectoral interventions to prevent, control and contain the virus. Because Ethiopia faces critical IPC challenges, the CDC Foundation, the International Center for AIDS Care and Treatment Program (ICAP) at Columbia University, and the Ethiopia Ministry of Health partnered to identify priority activities and health facilities to support with grant funding.

Through the project, designed to strengthen IPC programmes at different health systems levels, 16 Ethiopian healthcare facilities were chosen to receive IPC guidance and assistance, receiving technical assistance and financial support for baseline capacity assessments to understand their COVID-19 prevention, containment and management readiness. The project led to the design and development of IPC standardised operating procedures, including screening and identifying healthcare workers suspected of COVID-19 exposure. ICAP developed guidance documents to enable healthcare workers to establish effective and quality IPC practices, and all 16 facilities now have IPC operational plans and standardised procedures in place, including source control screening, triage for patients, visitors, and healthcare workers, and inpatient isolation.

Through the project, all 16 facilities helped develop national IPC indicators and dashboards to share across various levels of health care and strengthen cross-system collaboration; they now regularly report IPC indicators to the Ministry of Health. By identifying and addressing IPC programme gaps and improving capacity, this project also helped with the development and dissemination of a standardised programme that is now informing public health decisions via streamlined collection and reporting to the Ministry of Health.

## Case study: IPC assessment and healthcare workforce training in Indonesia

The burden of COVID-19 in Indonesia significantly exceeded the capacity of the country’s healthcare workers and contact tracers. While safe IPC was already a criterion to accredit Indonesia’s healthcare facilities, an intra-action review conducted by the Ministry of Health during the COVID-19 pandemic recommended improving IPC strategies throughout the country [10]. To reduce COVID-19 transmission, the CDC Foundation partnered with the Indonesian Epidemiological Association (PAEI) to provide training and technology and technical support to the epidemiological workforce in eight selected districts across four provinces.

**Figure 1:**
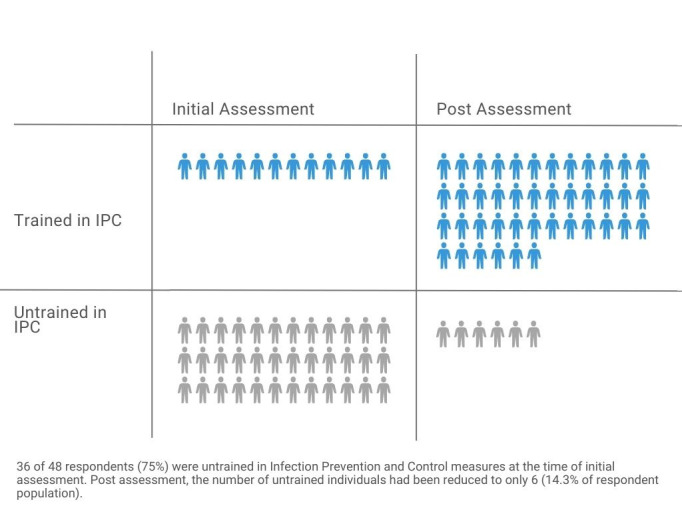
Healthcare workers in Indonesia trained on COVID-19 IPC based on initial and post-assessment, 2020.

An initial assessment of surveillance and IPC activities at 12 hospitals evaluated IPC implementation and training of healthcare workers during the COVID-19 pandemic, including epidemiology education and qualifications, types of training and site status of contact tracing. At the start of the project, only 12 out of the 48 total assessment respondents (25%) were trained in IPC (**Figure 1**). In a follow-up assessment in the four chosen districts, healthcare providers in hospitals reported that most staff (72%) had not received epidemiological surveillance training and only 28% had received any surveillance training. Additionally, only 58% of health offices within the four provinces had been conducting COVID-19 contact tracing programmes.

PAEI conducted training and held regular monitoring and evaluation meetings to improve surveillance and IPC activities in all the districts within the four provinces, training surveillance staff on reporting daily COVID-19 cases and field investigation results. During the project period, seven IPC training sessions were held, hosting 498 participants from 38 hospitals across the four provinces.

At provincial and district health offices, the number of staff trained in surveillance activities increased from 41% to 98% after the project; at community health centres, the number of staff trained in surveillance, contact tracing, and epidemiological investigation rose from 15% to 80%. The number of healthcare workers trained in IPC increased from 25% to 86% by the end of this five-month project (**Figure 1**). The number of hospitals with standard procedures for monitoring healthcare workers’ COVID-19 status increased from 58% to 83%, and the number that provided staff with regular COVID-19 testing increased to 100%.

## DISCUSSION

COVID-19 transmission rates exceeded healthcare capacity limitations and disproportionately impacted international healthcare systems. The Ethiopia and Indonesia case studies enhanced COVID-19 surveillance and detection by creating local, regional, and national systems and tools, improving testing capacity, providing IPC training for healthcare professionals, and bolstering COVID-19 mitigation efforts.

**Figure:**
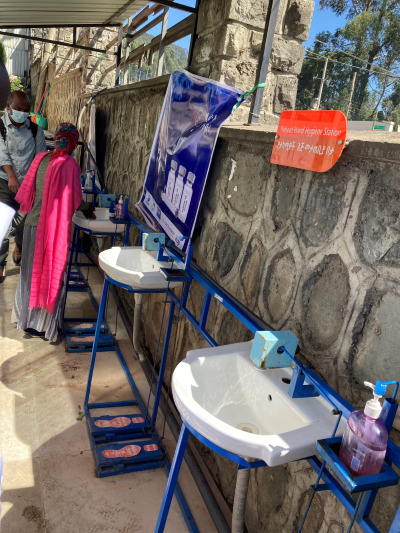
Photo: Patient handwashing stations installed at a healthcare facility in Addis, Ababa Ethiopia as part of the COSMIC project. Source: CDC Foundation, used with permission obtained from partners.

Going forward, countries should consider establishing national IPC guidelines and engaging local health facilities to create more sustainable responses, as collaboration between local facilities, implementing partners, and health authorities led to national IPC policies that were, in turn, adopted locally. Each programme case study also used flexible funding to meet the community’s needs and focused on embedding IPC programmes into both the local and national healthcare systems. Multi-sector collaboration between local and national experts around IPC programming created more resilient health systems through holistic and integrated approaches. By increasing capacity and training healthcare workers in IPC, countries can be better equipped to provide surveillance of future healthcare needs.
